# Enhanced spontaneous functional connectivity of the superior temporal gyrus in early deafness

**DOI:** 10.1038/srep23239

**Published:** 2016-03-17

**Authors:** Hao Ding, Dong Ming, Baikun Wan, Qiang Li, Wen Qin, Chunshui Yu

**Affiliations:** 1 School of Medical Imaging, Tianjin Medical University, Tianjin 300070, People’s Republic of China; 2 Department of Biomedical Engineering, Tianjin University, Tianjin 300072, People’s Republic of China; 3 Department of Radiology and Tianjin Key Laboratory of Functional Imaging, Tianjin Medical University General Hospital, Tianjin 300052, People’s Republic of China; 4 Technical College for the Deaf, Tianjin University of Technology, Tianjin 300384, People’s Republic of China

## Abstract

Early auditory deprivation may drive the auditory cortex into cross-modal processing of non-auditory sensory information. In a recent study, we had shown that early deaf subjects exhibited increased activation in the superior temporal gyrus (STG) bilaterally during visual spatial working memory; however, the changes in the organization of the STG related spontaneous functional network, and their cognitive relevance are still unknown. To clarify this issue, we applied resting state functional magnetic resonance imaging on 42 early deafness (ED) and 40 hearing controls (HC). We also acquired the visual spatial and numerical n-back working memory (WM) information in these subjects. Compared with hearing subjects, the ED exhibited faster reaction time of visual WM tasks in both spatial and numerical domains. Furthermore, ED subjects exhibited significantly increased functional connectivity between the STG (especially of the right hemisphere) and bilateral anterior insula and dorsal anterior cingulated cortex. Finally, the functional connectivity of STG could predict visual spatial WM performance, even after controlling for numerical WM performance. Our findings suggest that early auditory deprivation can strengthen the spontaneous functional connectivity of STG, which may contribute to the cross-modal involvement of this region in visual working memory.

Sensory deprivation during early age may drive the functional reorganisation of the deprived sensory cortex to process information from the remaining intact sensory modalities. Abundant evidence has demonstrated that the visual cortex of early blind subjects is involved in both tactile and auditory perceptual tasks^1^,^2^,^3^,^4^,^5^,^6^, and that the auditory cortex of early deaf (ED) subjects responds to visual and tactile perceptual stimuli^7^,^8^,^9^,^10^,^11^,^12^,^13^. Besides the cross-modal perceptual processing, the early deprived sensory areas are also involved in multiple high-level cognitive tasks, such as the visual regions in processing nonvisual memory, attention, imagery and language tasks in early blind subjects^2^,^14^,^15^,^16^,^17^, and the auditory regions in processing sign language^18^,^19^, visual attention^20^ and visual working memory (WM)^21^ in ED subjects. As a particular perceptual or cognitive task usually requires the synergism of distributed brain areas that constitute a complex network^22^,^23^, it is interesting to clarify whether the organizations of the intrinsic functional network of the reorganized sensory areas are also reshaped in adaptation to other sensory stimuli or top-down inputs.

The intrinsic functional network can be investigated via resting-state functional connectivity (FC) analysis, which measures the temporal correlation between time series of the spontaneous blood oxygen level–dependent (BOLD) signals of each pair of brain regions. Based on the patterns of FC strength among brain regions, we can identify many resting-state networks (RSN), such as the somatomotor^24^, visual^25^ and auditory^26^ networks, default mode network^27^, and several task-positive networks^28^, etc. Furthermore, the spatial patterns of the RSN are highly coherent with those of task-evoked brain networks^29^. The strength of FC of the brain network is correlated with the known anatomical systems^27^,^30^. Moreover, the inter-individual variability in FC strength relates to many types of behavioral performance and neurological and psychiatric disorders^31^,^32^,^33^,^34^. For example, the inter-individual differences in resting-state FC are closely correlated with the BOLD activity induced by WM tasks^35^, and the strength of FC can also predict the individual WM performance^36^,^37^. Early visual deprivation can reshape the FC of the occipital cortex, such as decreased FC between the occipital cortex and the nonvisual sensory cortices^38^,^39^,^40^,^41^,^42^ and increased FC between the occipital cortex and the prefrontal cortex in the early blind^38^,^40^,^43^. The cross-modal plasticity of the auditory cortex has been shown in the ED subjects^18^,^19^,^20^,^21^. However, it is not clear whether the resting state FC of the auditory cortex is also altered after early acoustic deprivation. Furthermore, it is still unknown whether the altered FC (if it is indeed altered) has behavioral significance.

In this study, we aimed to clarify the reorganization of the spontaneous functional network of superior temporal gyrus (STG) and their cognitive relevance in ED subjects using whole brain FC analyses. In a recent study, we had shown that ED subjects exhibited increased activation in the STG bilaterally during visual spatial WM with a right-side domination; furthermore, the increased activation amplitude of the STG predicts better WM performance in deaf subjects, indicating a cross-modal plasticity of the STG in visual WM processing^21^. Thus, we predict that the resting-state FC between these STG seeds and WM related brain regions are strengthened after early auditory deprivation. We also expected a significant correlation between the resting-state FC of STG and WM performance of several types of WM tasks.

## Materials and Methods

### Participants

Forty-two right-handed ED adults (23 females and 19 males, age range 20–26 years, the onset age of deafness within 2 years) were recruited from the Technical College for the Deaf of the Tianjin University of Technology in this study. Forty right-handed healthy hearing controls (20 females and 20 males, age range 20–26 years) were also enrolled. The detailed demographic information of these subjects was shown in Table 1. The hearing thresholds for all of the ED subjects were greater than 90 dB, which satisfied the criteria for profound deafness^44^. All of the 42 ED subjects had a history of hearing aid usage from 1.5–21 years after birth. All individuals had normal visual acuity and no history of neurological or psychiatric disorders. This study was approved by the Medical Research Ethics Committee of Tianjin Medical University General Hospital, and the study methods were conducted in accordance with the approved guidelines. All participants provided informed consent prior to the experiment.

### Imaging data acquisition

MRI data were acquired by a 3.0 Tesla MR system (Discovery MR750, General Electric, Milwaukee, WI, USA). The heads of subjects were stabilized by tight but comfortable foam padding to minimize head motion, and earplugs were used to reduce scanner noise. Sagittal 3D T1-weighted structural images were acquired using the brain volume (BRAVO) sequence with the following parameters: repetition time (TR) = 8.2 ms; echo time (TE) = 3.2 ms; inversion time (TI) = 450 ms; flip angle (FA) = 12°; field of view (FOV) = 256 mm × 256 mm; matrix = 256 × 256; slice thickness = 1 mm, and 188 continuous sagittal slices. The resting-state functional magnetic imaging (fMRI) data were obtained using the gradient-echo single-shot echo planar imaging (GRE-SS-EPI) sequence: TR/TE = 2000/45 ms; FOV = 220 mm × 220 mm; matrix = 64 × 64; FA = 90°; slice thickness = 4 mm; gap = 0.5 mm; 32 interleaved transverse slices, and 180 volumes. During the fMRI scan, all participants were instructed to keep awake with eyes closed and heads static, and to think nothing in particular.

### Visual spatial and numeric working memory tests

To evaluate the individual variance in visual working memory performance, immediately after the resting state fMRI scanning, all participants were informed to perform four conditions of n-back WM tests, including the visual spatial 1- and 2-back tasks (which measure the individual variability in spatial working memory ability) and numeric 1- and 2-back tasks (which measure the individual variability in verbal working memory ability). During the visual spatial n-back WM conditions, 25 squares (2.4° of visual angle for each) with white border were equally arranged in a 5 × 5 matrix and presented in the centre of the screen on a black background, in which only 1 square was randomly filled with white colour for each presentation. Participants were instructed to memorise the locations of the target square with white colouring, and press the response button using the right index finger once the location of the current target was same as the location of the one presented n trials previously. During the visual numerical n-back WM conditions, one decimal number (from 1–4, 5° of visual angle) with white colour is randomly presented in the centre of the screen on a black background. Participants were instructed to press the response button using the right index finger once the current number is same as the one presented n trials previously. All tasks were designed and presented by E-prime 2.0 (NordicNeuroLab, NNL). Each stimulus was displayed for 500 ms with an inter-stimulus interval of 2500 ms. Each of the four conditions consisted of 60 trials, with a 60-s inter-condition rest period. Besides the n-back WM task, we also collected the behavioural data from a visual spatial delayed recognition task during another task fMRI scan that had been reported in a recent study of our group^21^, which included four conditions with different setsize-loading combinations, i.e.: 4-1, 4-3, 12-1, 12-3. It should be noted that one ED subjects in this study did not perform the visual spatial delayed recognition task. For details of the experiment design please see the materials and methods section of the early study^21^.

The accuracy ratio and average reaction time (RT) of each experimental condition of each task in each subject were measured. To eliminate potential speed vs. accuracy trade-offs, inverse efficiency (IE) scores were also calculated by dividing the average RT by the accuracy ratio, which represents the corrected reaction time^45^,^46^.

### FMRI data preprocessing

The resting state fMRI data were preprocessed using a self-developed software based on the Statistical Parametric Mapping version 8 (SPM8; http://www.fil.ion.ucl.ac.uk/spm). The first 10 volumes for each subject were firstly discarded to remove the potential fMRI signals dropping caused by incomplete T1 relaxation, and allow the participants to adapt to the scanning environment. The remaining volumes were then corrected for the acquisition time delay between slices. And then, a rigid spatial realignment was carried out to correct for head motion among scan volumes. One male hearing subject was excluded because of his head motion exceeded the predefined motion threshold with translational motion of 2 mm or rotation of 2°. Considering recent studies reported that signal spikes caused by head motion can significantly contaminate the final resting state fMRI results, we also calculated the frame-wise displacement (FD), which represents the volume-by-volume head motion amplitude^47^. Then the six motion parameters and their first time derivatives, the volumes with FD exceeded 0.5, and the average BOLD signals of the ventricular, white matter and global brain tissue were regressed out from the fMRI data. The regressed fMRI data were subsequently band-pass filtered with a frequency range of 0.01–0.08 Hz to remove high-frequency noises (caused by respiratory and cardiac cycling, etc.) and low-frequency signal drifts^24^,^25^,^48^. Then the mean fMRI images (generated at the realignment step) of each subject were linearly coregistered with his/her structural images and the structural images were linearly coregistered to the T1 template of the MNI space using 12-parameters affine transformation. The transformation parameters of these two coregistration steps were then used to write the filtered fMRI data into MNI space, which were further resampled into voxel size of 3 × 3 × 3 mm^3^. Finally, the normalised fMRI data were smoothed with a Gaussian kernel of 6 × 6 × 6 mm^3^ full width at half maximum. To determine whether global brain signal regression (GSR) would influence the FC statistics^49^,^50^,^51^, we also performed a separate preprocessing step on the fMRI data with the same parameters but without regressing out the average BOLD signals of the global brain tissue.

### Computation of functional connectivity

The seeds of FC analyses were defined based on one of our former studies that investigated the cross-modal activation of auditory regions during visual spatial working memory in early deafness^21^. The regions of interest (ROIs) of the bilateral STG were extracted from the activation differential maps between the ED and HC at the WM maintenance stage. Voxels with increased activation (P < 0.05, FWE corrected) and that were within a 9-mm radius sphere centred on the peak of activation (left STG: [−51, −33, 6]; right STG: [63, −21, 3]) were defined as the STG seeds. The mean time series of BOLD signals of the bilateral STGs were then extracted and the Pearson correlation coefficients between the time series of the seed ROIs and that of each voxel of the whole brain gray matter were computed.

Because the variance of correlation coefficient (r) grows smaller as |r| gets closer to 1, which means that the population r is not normally distributed and the r values cannot be directly used for parametric test. Thus, a Fisher transformation algorithm was introduced to convert the correlation coefficient r of each connection to z value^52^,^53^. After Fisher transformation, the derived z values are approximately normally distributed while preserves the relative strength of correlation coefficients^54^, which were used for the following statistical analyses.

### Statistical analyses

A two-sample t-test was used to test the inter-group differences in RT and IE scores of visual WM and age. A Mann–Whitney U test was used for comparison of the accuracy ratio of visual WM between the ED and HC (q < 0.05, false discovery rate [FDR] corrected). A Chi-square test was used to assess the inter-group differences in gender (P < 0.05, uncorrected). Individuals’ z-transformed FCs of each STG of each group were firstly tested in a voxel-wise manner using a random effect one-sample t test in to identify cerebral grey matter regions that showed positive FC in this group (q < 0.05, peak topological FDR corrected for multiple comparison with peak threshold of P < 0.05). A two-sample t-test was then performed to compare the inter-group differences in FCs between the ED and HC controlling for age and gender effects within the masks that showed positive FCs either in the ED or HC (q < 0.05, peak topological FDR corrected). Then, the mean FC values of ROIs that showed altered FC in the ED were extracted, and a Pearson correlation coefficient analysis was carried out to investigate the associations between the FC values in these ROIs and the RT/IE scores, and a Spearman correlation coefficient analysis was used to test the correlation between the FC in these ROIs and accuracy ratio (q < 0.05, FDR corrected).

## Results

### Demographic and WM behavioural characteristics

As shown in Table 1, there was no significant difference in age (t = 0.764, P = 0.447) or gender (χ^2^ = 0.098, P = 0.754) between the two groups. Without using hearing aids, the mean hearing thresholds for the ED were 103.0 ± 9.5 dB in the left ear and 102.0 ± 8.6 dB in the right ear. The mean duration of auditory deprivation (including time when hearing aids were used) was 22.3 ± 1.8 years, the mean age of onset of hearing aid use was 11.7 ± 6.8 years, the mean percentage of lifetime hearing aid use was 51.2 ± 27.9%, the mean age of onset of sign language use was 6.8 ± 1.8 years, and the mean percentage of lifetime sign language use was 70.3 ± 8.0%.

**Figure 1:**
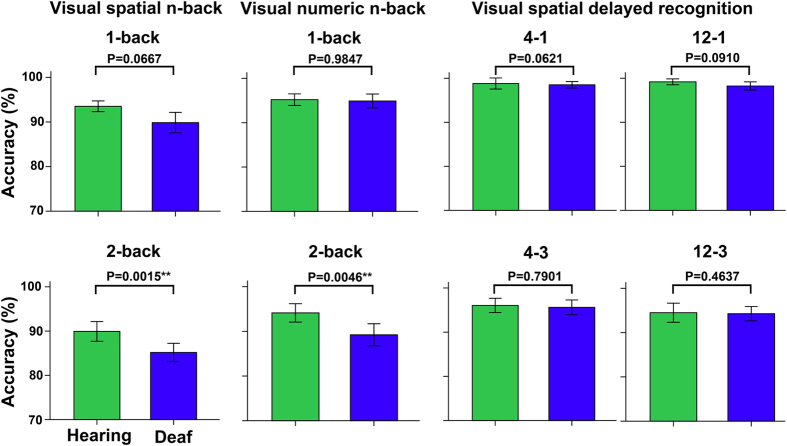
Differences in accuracy of working memory performance between the early deaf and hearing groups. A Mann–Whitney U test was used for comparison of the accuracy between the ED and HC. ** indicates statistical difference with P < 0.05 (FDR corrected). Compared with the HC, significant decreased accuracy was found when ED perform 2-back visual spatial and numeric tasks, while no statistical differences in the remaining behavior performance. ED = early deafness, FDR = false discovery rate, HC = sighted controls.

**Figure 2:**
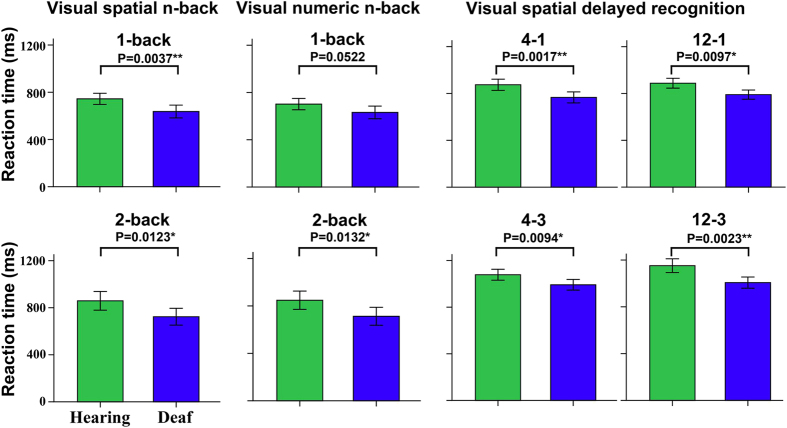
Differences in reaction time of working memory performance between the early deaf and hearing groups. A two-sample test was used for comparison of the reaction time between the ED and HC. ** indicates statistical difference with P < 0.05 (FDR corrected). * represents statistical difference with uncorrected P < 0.05. Compared with the HC, significant faster reaction time was found when ED perform 1-back spatial task, visual spatial delayed recognition task with setsize-load effect 4-1 and 12-3 (P < 0.05, FDR corrected), furthermore, a trend of faster reaction time was also found under the 2-back spatial and numeric tasks, setsize-loading effect of 12-1 and 4-3 in visual spatial delayed recognition task. ED = early deafness, FDR = false discovery rate, HC = sighted controls.

Compared with the HC, the ED exhibited significantly decreased accuracy when ED performed 2-back visual spatial and numeric tasks, while no statistical differences in accuracy of the remaining WM tasks (q < 0.05, FDR corrected) (Fig. 1). In contrast, the ED exhibited significantly faster RT and IE scores than the HC in most WM conditions (q < 0.05, FDR corrected, or uncorrected P < 0.05) (Fig. 2, Supplementary Fig. S1).

### FC patterns of the STG

**Figure 3:**
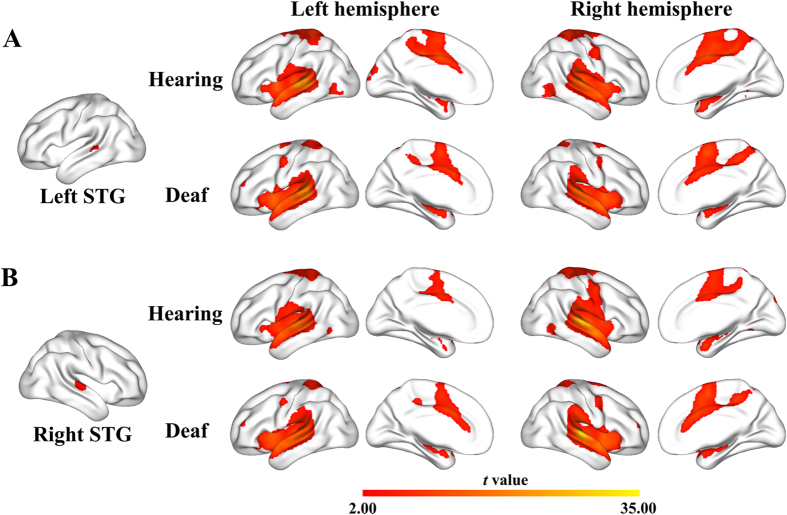
Functional connectivity patterns of the superior temporal gyrus. Both the hearing controls and early deaf subjects demonstrated similar positive functional connectivity of superior temporal gyrus (STG) with the bilateral superior temporal gyri and sulci, posterior and anterior insula, Rolandic operculum, postcentral gyrus, precentral gyrus, paracentral gyri, dorsal anterior cingulated cortex, and dorsolateral prefrontal cortex, most of which corresponded to the auditory network, salience network, and sensorimotor network (P < 0.05, peak-level topological FDR corrected).

As shown in Fig. 3, in the ED subjects, both the left and right STGs showed positive FC with bilateral superior temporal gyri and sulci, posterior and anterior insula, Rolandic operculum, postcentral gyri, precentral gyri, paracentral gyri, dorsal anterior cingulated cortices (dACC), and dorsolateral prefrontal cortices (dlPFC), most of which corresponded to the auditory network, salience network, and sensorimotor network. The HC exhibited an FC distribution similar to the ED, except that the HC showed additional positive FC between the STGs and middle occipital temporal (MOT) conjunction and cuneus, while missed positive FC between the STGs and dlPFC. It should be noted that we did not identify any positive FC between the STG seeds and visual cortex in the ED, which may not explain how the visual information reach the auditory cortex to drive cross-modal process. One possible explanation is that the auditory seeds we defined are not the hubs that connect the visual cortex in the ED. To clarify this issue, we introduced a voxel-wise visual-auditory functional connectivity density (FCD) analyses. This method was to calculate the number of functional connections between each auditory voxel and the visual cortex, and then identify core auditory hubs that have dense connections with the visual cortex (For detail processing information please see the Supplementary Materials). As seen in Supplementary Fig. S2, we found that the bilateral posterior STG (pSTG) had higher FCD (than mean value of the whole auditory cortex) with the visual cortex. Furthermore, the FC seeds used in this study were not overlapped with these hubs, indicating visual information may be not directly transferred to the seed region, but via the neighboring pSTG hubs. Further FC analysis identified a positive FC between the pSTG hubs and MOT regions (one sample t-test, P < 0.05 topological FDR corrected), indicating MOT- pSTG may be a candidate a pathway for conveying visual information to auditory cortex. Finally, we did not found a significant correlation between the strength of MOT-pSTG FC and working memory performance (P > 0.5), indicating that the MOT-STG connectivity may be more likely a general route for visual transformation rather than WM processing.

### Differences in FC of the STG between the deaf and hearing subjects

**Figure 4:**
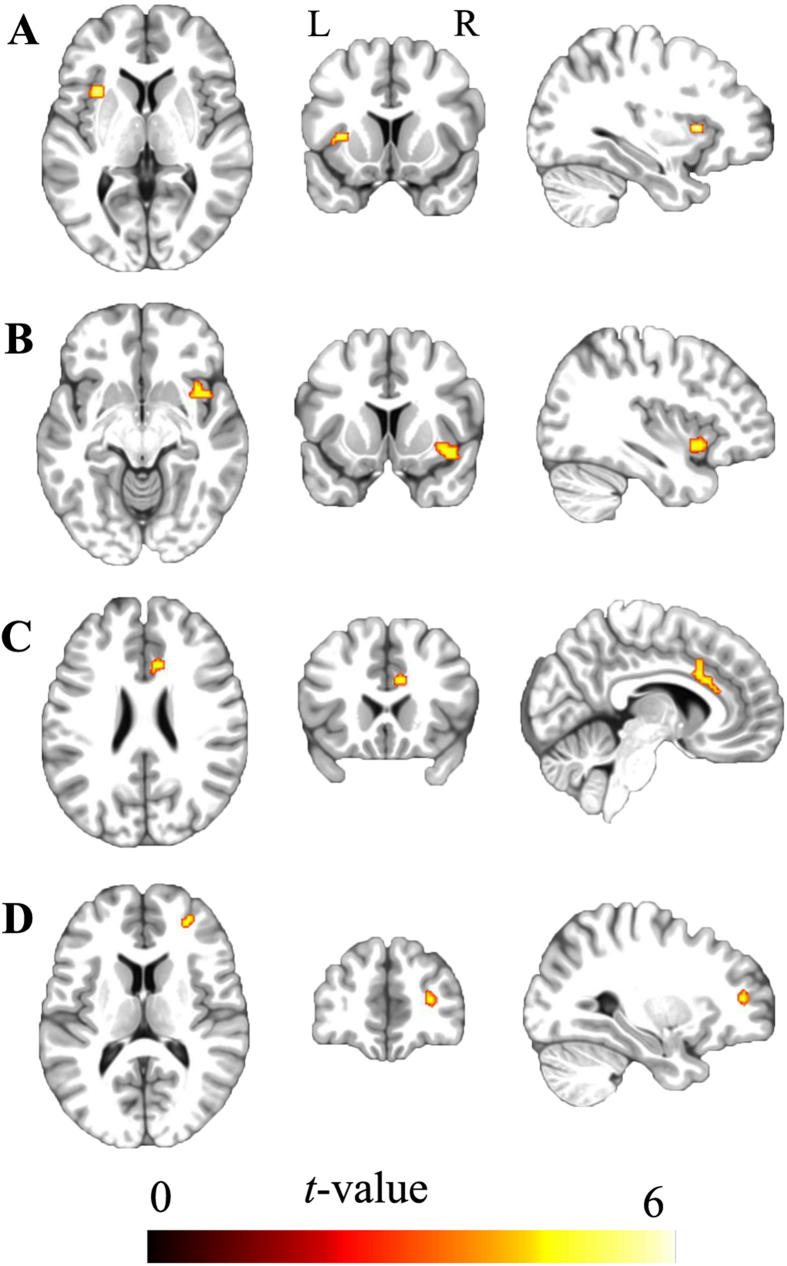
Differences in the functional connectivity of the right superior temporal gyrus between the early deaf and hearing groups. Compared with the hearing controls, the early deaf subjects exhibited significantly increased functional connectivity between the right superior temporal gyrus and (**A**) left anterior insula, (**B**) right anterior insula, (**C**) dorsal anterior cingulated cortex (**D**) and right dorsolateral prefrontal cortex (P < 0.05, peak-level topological FDR corrected).

After controlling for age and gender effects, the ED subjects exhibited significantly increased FCs between the right STG and bilateral anterior insula (aINS), dACC and right dlPFC (P < 0.05, topological FDR corrected) (Fig. 4). Using a looser threshold (P < 0.001, uncorrected), we also identified increased FC between the left STG and the bilateral aINS, dACC and left STG in the ED (Supplementary Fig. S3), indicating a similar alteration patterns as the right STG. To test whether these findings result from GSR in the data preprocessing step, we also compared the group differences in FC of STG that calculated based on fMRI data without GSR. Significantly increased FC was also identified in the ED compared with the HC in these brain regions (Supplementary Fig. S4).

### Correlations between FC of the STG and WM performance

Spearman correlation analysis did not identify significant correlation between the FCs of STG seeds and accuracy ratio of WM tasks (q < 0.05, FDR corrected), except a trend of negative correlation between the FCs in the left dACC (P = 0.031, uncorrected), left anterior STG (P = 0.020, uncorrected) and right dACC (P = 0.019, uncorrected) and the accuracy ratio of Numeric 1-back task (Supplementary Tables S1 and S2). In contrast, Pearson correlation analysis demonstrated significant negative correlations (q < 0.05, FDR corrected), or a trend of negative correlations (P < 0.05 uncorrected) between the RT and most ROIs that showed strengthened FC with STG after auditory deprivation (Table 2 and Supplementary Table S3). To eliminate potential speed vs. accuracy trade-offs, Pearson correlation analysis was also performed to test the association between corrected RT (that is, the IE scores) and FC of STG. Significant negative correlations (q < 0.05, FDR corrected), or a trend of negative correlations (P < 0.05 uncorrected) were still identified between the IE scores and most ROIs (Supplementary Tables S4 and S5), indicating that the FCs of the right STG are closely associated with the RT of visual WM performance. Since there were significant correlations between spatial and numeric RT(IE) scores in both the 1-back conditions and 2-back conditions, only based on the former correlation findings, we could not infer whether the FCs of STG are preferably associated with spatial or numeric WM performance. Consequently, additional partial correlation analyses were performed. After controlling for the numeric RT(IE) scores, the correlations between the spatial RT(IE) scores and the FC of the right STG still survived in most brain regions (P < 0.05, uncorrected). In contrast, the correlations between the numeric RT(IE) scores and the FC of the right STG never survived after controlling for the spatial RT(IE) scores (Table 3 and Supplementary Table S6). These findings suggest that the FCs of STG might be primarily associated with the spatial WM performance rather than the numeric one.

### Correlations between FC of the STG and clinical parameters

The correlations between the resting-state FC with right STG and clinical parameters in deaf subjects were shown in Supplementary Table S7. After controlling for gender and age effects, no correlation was observed between FCs of the STG and deafness duration, the age of onset of hearing aid use, or the age of onset of sign language (P > 0.05). In addition, after controlling for gender, no correlation were observed between FCs of the STG and the percentage of lifetime hearing aid use, or the percentage of lifetime of sign language (P > 0.05).

## Discussion

In this study, we used whole brain resting state FC analyses to investigate the alterations in spontaneous functional network of the STG in ED subjects, and to investigate the potential associations between the FC of STG and the WM performance. Compared with hearing subjects, we observed superior visual WM performance in the ED. Furthermore, significantly increased FCs were identified between the STG and brain regions that are primarily ascribed to the salience network. Finally, the FCs of the STG can predict visual spatial WM performance, even after controlling for numerical WM performance. Combined with an early study that reported cross-modal involvement of this region in processing visual spatial WM tasks^21^, our findings suggest that early auditory deprivation can not only enhance the regional activity of STG, but also strengthen the spontaneous functional organisation of STG network, which may contribute to the cross-modal involvement of this region in visual working memory.

In a previous study, we had shown that ED subjects exhibited faster visual spatial WM abilities using a delayed recognition task^21^. In this study, we further identified that the ED exhibited faster processing efficiency (as reflected by uncorrected and corrected reaction time) using n-back WM paradigms. Our findings were consistent with other studies that also reported superior visual WM performance for spaces, faces and shapes in the ED relative to the HC^55^,^56^,^57^. To elucidate the neural substrates of the compensatory WM abilities in the ED, in an early study, we also observed increased activation in the posterior superior temporal gyrus (STG) bilaterally during visual WM, and the increased activation amplitude of STG predicted faster visual spatial WM performance in deaf subjects^21^. This finding provided the functional specialization (functional segregation) of the posterior STG in cross-modal processing visual spatial WM tasks. Since a particular perceptual or cognitive process usually requires the synergism of distributed brain areas that constitute a complex network (functional integration)^22^,^23^, it is interesting to elucidate whether the organizations of the spontaneous functional network of the reorganized STG are also reshaped to compensate the sound loss. In this study, it is interesting to note that the STG, as identified by an early WM activation study, showed significantly enhanced resting state FC with the brain regions that are mainly ascribed to the hubs of the salience network in the ED, and the increase in FCs were significantly associated with faster visual WM processing. Our findings provided additional information about the neural substrates of the cross-modal plasticity of the auditory areas in the ED: the enhanced visual spatial WM abilities in the ED might be the consequence of neural plasticity in both functional segregation and integration domains. Specifically, they are not only driven by enhanced activity of regional auditory areas (functional segregation domain), but also the enhanced functional coupling of the reshaped associated auditory areas with the remote hubs they connect to (functional integration domain).

Notably, bilateral STG in both ED and HC subjects are strongly functionally connected with the salient network, and strengthened FC of STG in the ED were also located in the core hubs of the salient network, such as the anterior insula and dACC. As core hubs of the salience network, the anterior insula primarily serves to identify behaviourally relevant but salient stimuli from sensory modalities and then forwards them to the central executive network to mediate attention, working memory and other higher order cognitive processes^58^. While the dACC plays a more prominent role in modulating responses to these stimuli, such as action selection or conflict solving^59^. Our findings provide the first evidence of the increased functional coupling between deprived auditory regions and salient network in early deaf subjects. these findings were consistent with early findings that showed increased FC within the salient network and between the salient and fronto-parietal networks in the CB^60^, and reduced regional spontaneous brain activity in the salient network in both the CB and LB^61^. Our findings were also consistent with one study that reported a stronger activation of the anterior insula and dACC during the short-term verbal memory task in early deaf signer compared with the hearing speaker^62^. The enhanced FC of the salient network may be a general consequence in response to early sensory deprivation, which facilitates the identification of salient stimuli from the deprived sensory areas (and may also from other intact sensory modalities), and then contributes to the enhanced attentional and working memory abilities.

The STG seeds used for calculating the FCs in the present study were based on a recent visual spatial WM activation study, in which a right-side domination of the activation enhancement of the STG was identified in the ED^21^. In this study, we also found that the right STG showed stronger augmentation in FC compared with the left side in the ED. Furthermore, brain regions showing increased FC in the right STG were predominantly located in the right hemisphere. In hearing subjects, lateralisation is an important property for the brain functions. For example, the left brain is preferred for language processing^63^,^64^, while the right brain is dominant in spatial processing^65^,^66^,^67^. Several early studies also demonstrated that this functional lateralization is also preserved in the deprived auditory areas. For example, in early deaf subjects, sign language can evoke greater activation in the left auditory areas^68^,^69^,^70^, while visual motion and spatial processing can evoke greater activation in the right auditory areas^8^,^70^,^71^. Consequently, the right-side lateralization of our findings suggests that the spontaneous functional network of STG in the ED may be associated with spatial function.

Because the spontaneous fMRI data are obtained without performing any specific tasks during the scan, it would be difficult to relate their derived FC measures to a certain brain function if no behavior data are collected. Under this situation, it would be impossible exclude the possibility that and the enhanced FC of STG in the ED is just a general phenomenon of reorganisation in spontaneous functional network following auditory deprivation. To further elucidate the functional preference of the spontaneous functional network of STG, we introduced three types of visual WM tasks (including the spatial delayed recognition tasks, spatial n-back tasks, and the numeric n-back tasks) and attempted to find possible associations between the FC of STG and the behavioural performance of these tasks. In accordance with our prediction, significant negative correlations were observed between the FC of STG and the RT(IE) scores of the three types of visual WM tasks, suggesting the increased FCs of STG are closely associated with visual WM performance. To further clarify whether the association between FCs of STG and WM performance is more preferably explained by spatial or numeric domain, we made additional correlation analyses by controlling for the effects of spatial or numeric tasks on each other. After controlling for the RT(IE) of numeric n-back conditions, we found that the correlations between the FCs of STG and the RT(IE) of spatial n-back conditions still survived; however, after controlling for the performance of spatial n-back conditions, the correlations between the FCs of STG and the performance of numeric n-back conditions never survived. Based on the findings of an early study that reported significant associations between the activation amplitude of STG and visual spatial WM performance in the ED^21^, and according to the right-side lateralization of the enhanced FC of STG in the ED, we suggest that the increased FC of STG in this study may be preferably associated with visual spatial WM processing, rather than being a general alteration without functional significance. Of course, we cannot infer that the strengthened FC of STG is specific for visual processing, especially under the situation that we had not acquired the behavior data from other sensory modality (such as somatosensory and olfaction). We also cannot infer that the strengthened STG FC is unique for working memory, because working memory is a general cognitive function that coexisted in many other perceptual and high cognitive processes (such as attention and discrimination), which have also been reported to evoke cross-modal activation in the STG of ED^20^,^68^,^72^,^73^. This topic will be interesting to cover in the future studies.

Finally, we did not identify any significant correlations between the FCs of STG and clinical parameters. These finding were not consistent with the early finding showing STG activation of the ED had positive correlation with the age of onset of hearing aid use, and negative correlation with the percentage of lifetime hearing aid use^21^. These findings may indicate that the enhanced functional connectivity between the salient network and STG is a general consequence in response to sensory deprivation and that early auditory experience may not contributed to normal development of this network. This hypothesis was supported by a recent study that showed that both the CB and LB demonstrated reduced regional spontaneous brain activity in the salient network^61^. However, this hypothesis needs to be verified in the future by additionally enrolling the late deaf subjects.

## Additional Information

**How to cite this article**: Ding, H. *et al*. Enhanced spontaneous functional connectivity of the superior temporal gyrus in early deafness. *Sci. Rep*. **6**, 23239; doi: 10.1038/srep23239 (2016).

## Supplementary Material

Supplementary Information

## Figures and Tables

**Table 1:** Demographic and clinical information of subjects.

	Hearing controls	Early deafness	Statistics	P value
Gender (males/females)	19/20	19/23	χ^2^ = 0.098	0.754
Age (years)	23.1 ± 1.2	22.9 ± 1.6	t = 0.764	0.447
Age of onset of deafness (years)	–	0.6 ± 0.7	–	–
Duration of deafness (years)	–	22.3 ± 1.8	–	–
Age of onset of hearing aid use (years)	–	11.7 ± 6.8	–	–
Percentage of lifetime hearing aid use (%)		51.2 ± 27.9		
Age of onset of sign language use (years)		6.8 ± 1.8		
Percentage of lifetime sign language use (%)		70.3 ± 8.0		
Degree of hearing loss (dB)				
Left ear	–	103.0 ± 9.5	–	–
Right ear	–	102.0 ± 8.6	–	–

**Table 2:** Correlations between functional connectivity of the right superior temporal gyrus and reaction time of working memory tasks.

	L_aINS		R_aINS		R_dACC		R_dlPFC	
	r	p	r	p	r	p	r	p
Spatial 1-back	**−0.274**	*0.013	**−0.358**	**<0.001	**−0.349**	**0.001	**−0.280**	*0.011
Spatial 2-back	**−0.333**	**0.002	**−0.398**	**<0.001	**−0.366**	**0.001	**−0.356**	**0.001
Numeric 1-back	−0.196	0.080	−0.139	0.215	−0.209	0.061	−0.131	0.242
Numeric 2-back	**−0.300**	**0.007	**−0.323**	**0.003	**−0.332**	**0.002	**−0.224**	*0.045
SDR 4-1	**−0.403**	**<0.001	**−0.281**	*0.012	**−0.361**	**0.001	**−0.243**	*0.030
SDR 4-3	−0.209	0.062	**−0.253**	*0.023	**−0.302**	**0.006	**−0.289**	*0.009
SDR 12-1	**−0.326**	**0.003	**−0.331**	**0.003	**−0.385**	**<0.001	**−0.235**	*0.036
SDR 12-3	**−0.315**	**0.004	**−0.360**	**0.001	**−0.374**	**0.001	**−0.246**	*0.028

** indicates significance survived under false discovery rate corrected q < 0.05. * indicates significance survived under uncorrected P < 0.05. Abbreviations: aINS = anterior insular; dACC = dorsal anterior cingulated cortex; dlPFC = dorsal lateral prefrontal cortex; L = left hemisphere; R = right hemisphere; SDR = spatial delayed recognition task.

**Table 3:** Correlations between functional connectivity of the right superior temporal gyrus and n-back reaction time after controlling for the effect of the other n-back domain.

	L_aINS		R_aINS		R_dACC		R_dlPFC		Controlling factor
	r	p	r	p	r	p	r	p	
Spatial 1-back RT	−0.195	0.083	**−0.372**	**0.001	**−0.291**	*0.009	**−0.268**	*0.016	Numeric 1-back RT
Spatial 2-back RT	−0.172	0.128	**−0.250**	*0.025	−0.188	0.095	**−0.293**	*0.008	Numeric 2-back RT
Numeric 1-back RT	−0.001	0.991	0.176	0.117	0.060	0.595	0.101	0.371	Spatial 1-back RT
Numeric 2-back RT	−0.079	0.484	−0.039	0.733	−0.093	0.412	0.071	0.531	Spatial 2-back RT

** indicates significance survived under false discovery rate corrected q < 0.05. * indicates significance survived under uncorrected P < 0.05. The r represents Pearson correlation coefficient. Abbreviations: aINS = anterior insular; dACC = dorsal anterior cingulated cortex; dlPFC = dorsal lateral prefrontal cortex; L = left hemisphere; R = right hemisphere; RT = reaction time; SDR = spatial delayed recognition task.
