# Supplementary material for: Enhanced spontaneous functional connectivity of the superior temporal gyrus in early deafness
Source: Sci Rep. 2016 Mar 17;6:23239. doi: 10.1038/srep23239 (PMC4794647; doi:10.1038/srep23239)
Supplement: Supplementary Information [file srep23239-s1.doc]

# Enhanced spontaneous functional connectivity of the superior temporal gyrus in early deafness

Hao Ding^1,2^, Dong Ming^2,*^, Baikun Wan^2^, Qiang Li^4^, Wen Qin^3,*^, Chunshui Yu^3^

^1^ School of Medical Imaging, Tianjin Medical University, Tianjin 300070, People’s Republic of China; ^2^ Department of Biomedical Engineering, Tianjin University, Tianjin 300072, People’s Republic of China; ^3^ Department of Radiology and Tianjin Key Laboratory of Functional Imaging, Tianjin Medical University General Hospital, Tianjin 300052, People’s Republic of China; ^4^ Technical College for the Deaf, Tianjin University of Technology, Tianjin 300384, People’s Republic of China

* Correspondence to: Wen Qin, Department of Radiology and Tianjin Key Laboratory of Functional Imaging, Tianjin Medical University General Hospital, Tianjin 300052, People’s Republic of China, E-mail: [wayne.wenqin@gmail.com](mailto:wayne.wenqin@gmail.com), Phone: +86-22-60363760，Fax: +86-22-60362290; or Dong Ming, Department of Biomedical Engineering, Tianjin University, Tianjin 300072, People’s Republic of China, E-mail: [richardming@tju.edu.cn](mailto:richardming@tju.edu.cn). Phone: +86-022-27408718, Fax: +86-022-27406726.

**Supplementary materials**

**Supplementary methods**

we introduced a voxel-wise visual-auditory functional connectivity density (FCD) analyses. This method was to calculate the number of functional connections between each auditory voxel and the visual cortex, and then identify core auditory hubs that have dense connections with the visual cortex. To perform this analysis, we first extract the visual cortex and auditory cortex based on the Harvard-Oxford Cortical Structural maximum probability maps (http://fsl.fmrib.ox.ac.uk/fsl/fslwiki/Atlases), in which the visual cortex includes the superior and inferior division of lateral occipital cortex, intracalcarine cortex, cuneal cortex, lingual gyrus, temporal occipital fusiform cortex, occipital fusiform gyrus, supracalcarine cortex, and occipital pole, and the auditory cortex included anterior and posterior division of superior temporal gyrus, planum polare, Heschl’s gyrus, planum temporale, and dorsal division of temporal pole. We then calculated the functional connectivity of each pair voxels between visual cortex and auditory cortex using the preprocessed fMRI data of this study. A threshold of P<0.05 (Bonferroni correction) was used to identify the positive FC. Then the positive functional connectivity matrix for each subject was extracted and binarized. The FCD of a certain auditory voxel was defined as the number of positive functional connections of this voxel with the visual cortex, vice versa.

To identify the hubs of auditory with the visual cortex, we first convert the FCD to z-score (zFCDi = [FCDi-meanFCD]/stdFCD, FCDi represents the FCD of each voxel, meanFCD represents the mean FCD of the total auditory voxels, stdFCD means the standard deviation of the FCD of the total auditory voxels). Then a one-sample t-test was used for identify brain regions with statistical positive zFCD values (P<0.05, corrected using topological FDR), which was considered as the hubs of auditory cortex with the visual cortex.

To identify which visual regions have positive FC with the auditory, we further calculated the FC between the posterior STG hubs and each voxels of the visual cortex (detail pipeline for FC calculation can be seen in the methods). Then a one-sample t-test was used for identify visual regions that have positive FC with the STG hubs (P<0.05, corrected using topological FDR).

**Supplementary Tables**

**Supplementary Table S1. Correlations between functional connectivity of the left superior temporal gyrus and accuracy** of working memory tasks.

|  | L_aINS | | R_aINS | | L_dACC | | L_STG | |
| --- | --- | --- | --- | --- | --- | --- | --- | --- |
|  | r | p | r | p | r | p | r | p |
| Spatial 1-back | 0.047 | 0.678 | 0.035 | 0.756 | -0.029 | 0.794 | -0.112 | 0.319 |
| Spatial 2-back | -0.043 | 0.704 | -0.035 | 0.758 | -0.103 | 0.359 | -0.166 | 0.138 |
| Numeric 1-back | -0.145 | 0.197 | -0.124 | 0.271 | **-0.241** | ***0.031** | **-0.257** | ***0.020** |
| Numeric 2-back | 0.021 | 0.854 | 0.056 | 0.617 | 0.017 | 0.884 | -0.099 | 0.378 |
| SDR 4-1 | 0.042 | 0.713 | 0.048 | 0.674 | 0.054 | 0.632 | 0.002 | 0.986 |
| SDR 4-3 | -0.176 | 0.118 | -0.183 | 0.105 | -0.127 | 0.260 | -0.129 | 0.253 |
| SDR 12-1 | -0.023 | 0.837 | 0.074 | 0.514 | 0.040 | 0.724 | -0.041 | 0.715 |
| SDR 12-3 | -0.005 | 0.966 | -0.063 | 0.578 | -0.097 | 0.392 | -0.025 | 0.829 |

* indicates significance survived under uncorrected P<0.05. Abbreviations: aINS = anterior insular; dACC = dorsal anterior cingulated cortex; L = left hemisphere; R = right hemisphere; SDR= spatial delayed recognition task; STG = superior temporal gyrus.

**Supplementary Table S2. Correlations between functional connectivity of the right superior temporal gyrus and accuracy of working memory tasks.**

|  | L_aINS | | R_aINS | | R_dACC | | R_dlPFC | |
| --- | --- | --- | --- | --- | --- | --- | --- | --- |
|  | r | p | r | p | r | p | r | p |
| Spatial 1-back | -0.072 | 0.525 | -0.017 | 0.881 | -0.101 | 0.370 | -0.090 | 0.425 |
| Spatial 2-back | -0.112 | 0.318 | -0.177 | 0.114 | -0.212 | 0.057 | -0.077 | 0.492 |
| Numeric 1-back | -0.035 | 0.756 | -0.141 | 0.210 | **-0.261** | ***0.019** | -0.039 | 0.733 |
| Numeric 2-back | -0.157 | 0.163 | -0.131 | 0.244 | -0.179 | 0.110 | -0.139 | 0.217 |
| SDR 4-1 | -0.188 | 0.095 | -0.071 | 0.530 | -0.166 | 0.142 | -0.152 | 0.178 |
| SDR 4-3 | 0.145 | 0.199 | -0.109 | 0.336 | -0.163 | 0.148 | -0.123 | 0.277 |
| SDR 12-1 | 0.045 | 0.694 | -0.061 | 0.594 | -0.093 | 0.414 | -0.161 | 0.154 |
| SDR 12-3 | 0.041 | 0.717 | -0.035 | 0.755 | -0.035 | 0.760 | 0.046 | 0.684 |

* indicates significance survived under uncorrected P<0.05. Abbreviations: aINS = anterior insular; dACC = dorsal anterior cingulated cortex; dlPFC = dorsal lateral prefrontal cortex; L = left hemisphere; R = right hemisphere; SDR= spatial delayed recognition task.

**Supplementary Table S3. Correlations between functional connectivity of the left superior temporal gyrus and reaction time of working memory tasks.**

|  | L_aINS | | R_aINS | | L_dACC | | L_STG | |
| --- | --- | --- | --- | --- | --- | --- | --- | --- |
|  | r | p | r | p | r | p | r | p |
| Spatial 1-back | **-0.267** | ***0.016** | **-0.298** | ***0.007** | **-0.252** | ***0.023** | -0.209 | 0.061 |
| Spatial 2-back | **-0.258** | ***0.020** | **-0.254** | ***0.022** | **-0.241** | ***0.030** | -0.219 | 0.050 |
| Numeric 1-back | **-0.275** | ***0.013** | **-0.260** | ***0.019** | -0.201 | 0.072 | **-0.300** | ***0.006** |
| Numeric 2-back | **-0.264** | ***0.017** | -0.192 | 0.085 | -0.206 | 0.065 | **-0.268** | ***0.016** |
| SDR 4-1 | **-0.301** | ***0.007** | **-0.249** | ***0.026** | **-0.246** | ***0.028** | -0.178 | 0.115 |
| SDR 4-3 | **-0.370** | ****<0.001** | **-0.304** | ***0.006** | **-0.281** | ***0.012** | **-0.223** | ***0.047** |
| SDR 12-1 | **-0.335** | ***0.002** | **-0.307** | ***0.006** | **-0.317** | ***0.004** | **-0.265** | ***0.018** |
| SDR 12-3 | **-0.391** | ****<0.001** | **-0.396** | ****<0.001** | **-0.365** | ****<0.001** | **-0.276** | ***0.013** |

** indicates significance survived under false discovery rate corrected q<0.05. * indicates significance survived under uncorrected P<0.05. Abbreviations: aINS = anterior insular; dACC = dorsal anterior cingulated cortex; L = left hemisphere; R = right hemisphere; SDR= spatial delayed recognition task; STG = superior temporal gyrus.

**Supplementary Table S4. Correlations between functional connectivity of the left superior temporal gyrus and inverse efficiency of working memory tasks.**

|  | L_aINS | | R_aINS | | L_dACC | | L_STG | |
| --- | --- | --- | --- | --- | --- | --- | --- | --- |
|  | r | p | r | p | r | p | r | p |
| Spatial 1-back | **-0.250** | ***0.024** | **-0.276** | ***0.013** | **-0.226** | ***0.042** | -0.164 | 0.143 |
| Spatial 2-back | **-0.265** | ***0.017** | **-0.242** | ***0.030** | -0.195 | 0.081 | -0.175 | 0.117 |
| Numeric 1-back | **-0.244** | ***0.028** | **-0.235** | ***0.035** | -0.151 | 0.180 | **-0.255** | ***0.022** |
| Numeric 2-back | **-0.266** | ***0.016** | -0.203 | 0.069 | -0.208 | 0.062 | **-0.220** | ***0.049** |
| SDR 4-1 | **-0.324** | ***0.003** | **-0.269** | ***0.016** | **-0.266** | ***0.017** | -0.181 | 0.108 |
| SDR 4-3 | **-0.302** | ***0.007** | -0.205 | 0.068 | -0.172 | 0.128 | -0.147 | 0.192 |
| SDR 12-1 | **-0.323** | ***0.003** | **-0.300** | ***0.007** | **-0.295** | ***0.008** | **-0.244** | ***0.029** |
| SDR 12-3 | **-0.348** | ***0.002** | **-0.333** | ***0.003** | **-0.272** | ***0.015** | -0.202 | 0.072 |

** indicates significance survived under false discovery rate corrected q<0.05. * indicates significance survived under uncorrected P<0.05. Abbreviations: aINS = anterior insular; dACC = dorsal anterior cingulated cortex; L = left hemisphere; R = right hemisphere; SDR= spatial delayed recognition task; STG = superior temporal gyrus.

**Supplementary Table S5. Correlations between functional connectivity of the right superior temporal gyrus and inverse efficiency of working memory tasks.**

|  | L_aINS | | R_aINS | | R_dACC | | R_dlPFC | |
| --- | --- | --- | --- | --- | --- | --- | --- | --- |
|  | r | p | r | p | r | p | r | p |
| Spatial 1-back | **-0.257** | ***0.020** | **-0.324** | ****0.003** | **-0.306** | ***0.005** | **-0.248** | ***0.025** |
| Spatial 2-back | **-0.320** | ****0.004** | **-0.377** | ****0.001** | **-0.312** | ***0.005** | **-0.371** | ****0.001** |
| Numeric 1-back | -0.194 | 0.083 | -0.114 | 0.312 | -0.173 | 0.122 | -0.126 | 0.261 |
| Numeric 2-back | **-0.259** | ***0.019** | **-0.301** | ***0.006** | **-0.278** | ***0.012** | -0.193 | 0.085 |
| SDR 4-1 | **-0.400** | ****<0.001** | **-0.296** | ***0.008** | **-0.369** | ****0.001** | **-0.250** | ***0.025** |
| SDR 4-3 | **-0.247** | ***0.027** | -0.186 | 0.098 | -0.207 | 0.065 | **-0.226** | ***0.043** |
| SDR 12-1 | **-0.321** | ****0.004** | **-0.313** | ***0.005** | **-0.360** | ****0.001** | -0.212 | 0.059 |
| SDR 12-3 | **-0.310** | ***0.005** | **-0.351** | ****0.001** | **-0.342** | ****0.002** | **-0.284** | ***0.011** |

** indicates significance survived under false discovery rate corrected q<0.05. * indicates significance survived under uncorrected P<0.05. Abbreviations: aINS = anterior insular; dACC = dorsal anterior cingulated cortex; dlPFC = dorsal lateral prefrontal cortex; L = left hemisphere; R = right hemisphere; SDR= spatial delayed recognition task.

**Supplementary Table S6. Correlations between functional connectivity of the right superior temporal gyrus and n-back inverse efficiency** after controlling for the effect of the other n-back domain.

|  | L_aINS | | R_aINS | | R_dACC | | R_dlPFC | | Controlling factor |
| --- | --- | --- | --- | --- | --- | --- | --- | --- | --- |
|  | r | p | r | p | r | p | r | p |  |
| Spatial 1-back IE | -0.175 | 0.125 | **-0.366** | ***0.001** | **-0.280** | ***0.013** | 0.219 | 0.054 | Numeric 1-back IE |
| Spatial 2-back IE | -0.208 | 0.068 | **-0.254** | ***0.025** | -0.176 | 0.122 | **-0.337** | ***0.003** | Numeric 2-back IE |
| Numeric 1-back IE | -0.020 | 0.860 | 0.1750 | 0.126 | 0.0627 | 0.586 | 0.0512 | 0.656 | Spatial 1-back IE |
| Numeric 2-back IE | -0.053 | 0.640 | -0.069 | 0.546 | -0.100 | 0.384 | 0.096 | 0.405 | Spatial 2-back IE |

* indicates significance survived under uncorrected P<0.05. The r represents Pearson correlation coefficient. Abbreviations: aINS = anterior insular; dACC = dorsal anterior cingulated cortex; dlPFC = dorsal lateral prefrontal cortex; IE = inverse efficiency; L = left hemisphere; R = right hemisphere; RT = reaction time; SDR= spatial delayed recognition task.

**Supplementary Table S7. Correlations between functional connectivity of the right superior temporal gyrus and clinical parameters in early deaf subjects.**

|  | **Duration of deafness (years)** | **Age of onset of hearing aid use (years)** | **Percentage of lifetime hearing aid use (%)** | **Age of onset of sign language use (years)** | **Percentage of lifetime sign language use (%)** |
| --- | --- | --- | --- | --- | --- |
| **Left insula** | -0.0075 | -0.1416 | 0.0582 | -0.296 | 0.0312 |
|  | (0.9631) | (0.3835) | (0.7177) | (0.8563) | (0.8466) |
| **Right insula** | 0.0352 | -0.1248 | 0.2281 | 0.0936 | -0.0833 |
|  | (0.8293) | (0.4431) | (0.1516) | (0.5655) | (0.6046) |
| **Right dACC** | 0.3008 | -0.0692 | 0.1698 | 0.2721 | -0.2627 |
|  | (0.0593) | (0.6713) | (0.2884) | (0.0893) | (0.0971) |
| **Right dlPFC** | -0.0040 | 0.0348 | 0.1709 | 0.0116 | -0.0016 |
|  | (0.9805) | (0.8311) | (0.2854) | (0.9433) | (0.9922) |

Note: Partial correlation analysis controlled for age and gender was applied. Data are presented as correlation coefficient (P value). dACC =dorsal anterior cingulate cortex; dlPFC = dorsal lateral perfrontal cortex; IE = inverse efficiency.

**Spplementary Figures**


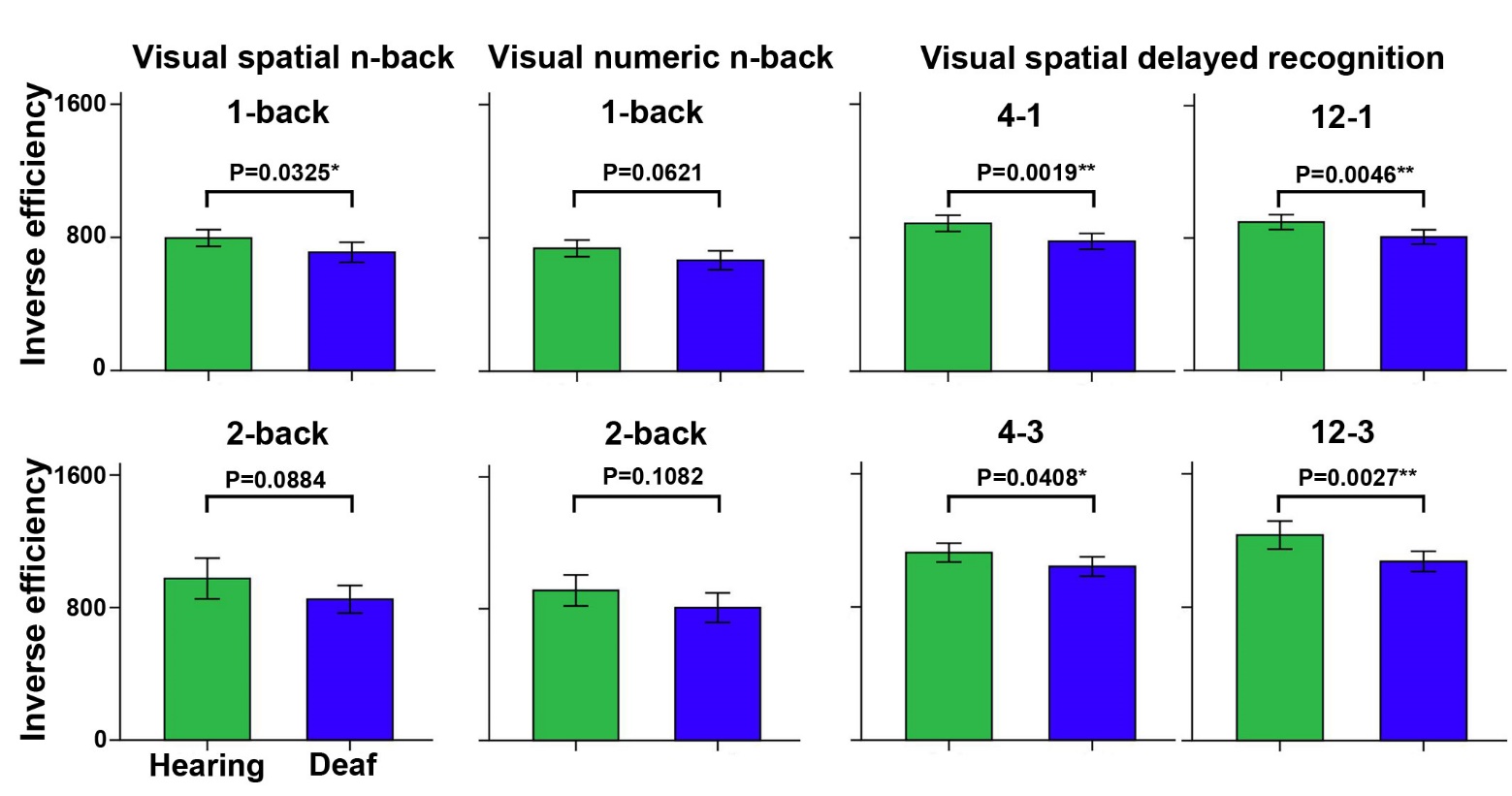


**Supplementary Figure S1**: Group differences in inverse efficiency of working memory performance. A two-sample test was used for comparison of the inverse efficiency between the ED and HC. ** indicates statistical difference with P <0.05 (FDR corrected). * represents statistical difference with uncorrected P <0.05. Compared with the HC, significant smaller inverse efficiency was found when ED perform visual spatial delayed recognition task with setsize-load effect 4-1, 12-1 and 12-3 (P<0.05, FDR corrected); furthermore, a trend of smaller inverse efficiency was also found under the 1-back spatial task and setsize-loading effect of 4-3 in visual spatial delayed recognition task. ED = early deafness, FDR = false discovery rate, HC = sighted controls.


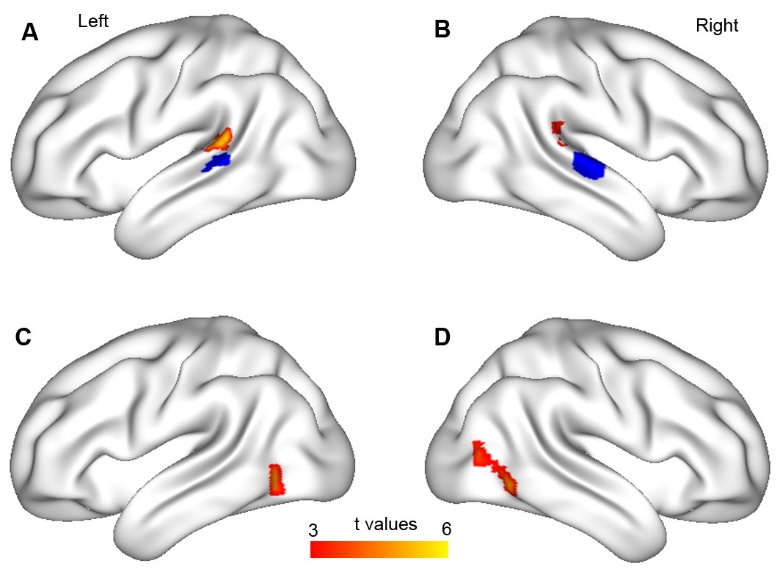


**Supplementary Figure S2: Functional connectivity between auditory and visual cortex in the ED.** The hot labels in Panel **(A)** and **(B)** represent the brain regions that have FCD above mean value of the whole auditory cortex (one sample t-test, P <0.05 topoFDR corrected). The blue labels represent the seed of STG for FC that derived from the cross-modal activation in ED in response for visual spatial delayed recognition task. The FC seed was not overlapped with the high FCD hubs, indicating visual information may be not directly transferred to the seed region, but more possibly via the neighboring auditory hubs. The hot labels in panel C and D represent visual regions that have positive FC with the auditory hubs (one sample t-test, P <0.05 topoFDR corrected). Abbreviations: ED = early deafness; FCD = functional connectivity density; FC =functional connectivity; FDR = false discovery rate; STG =superior temporal gyrus.


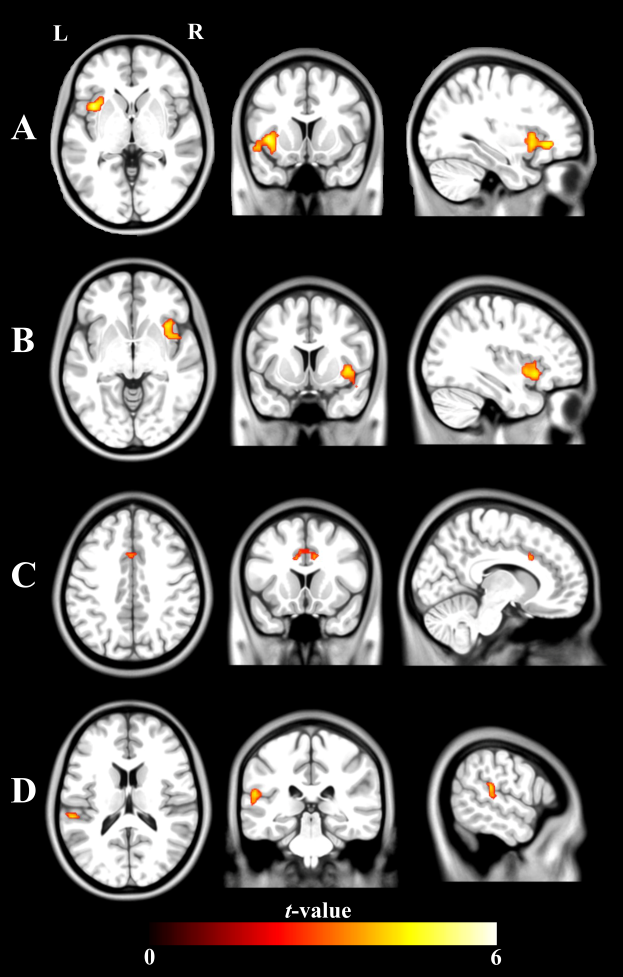


**Supplementary Figure S3. Differences in functional connectivity of left superior temporal gyrus between the early deaf and hearing groups.** After controlling for age and gender effects, the ED subjects exhibited significantly increased functional connectivity between the left STG left anterior insula **(A)**, right anterior insula **(B)**, dACC **(C)** and left STG **(D)** compared with the hearing control subject (P < 0.001,uncorrected). dACC =dorsal anterior cingulate cortex; ED = early deaf; STG =superior temporal gyrus.


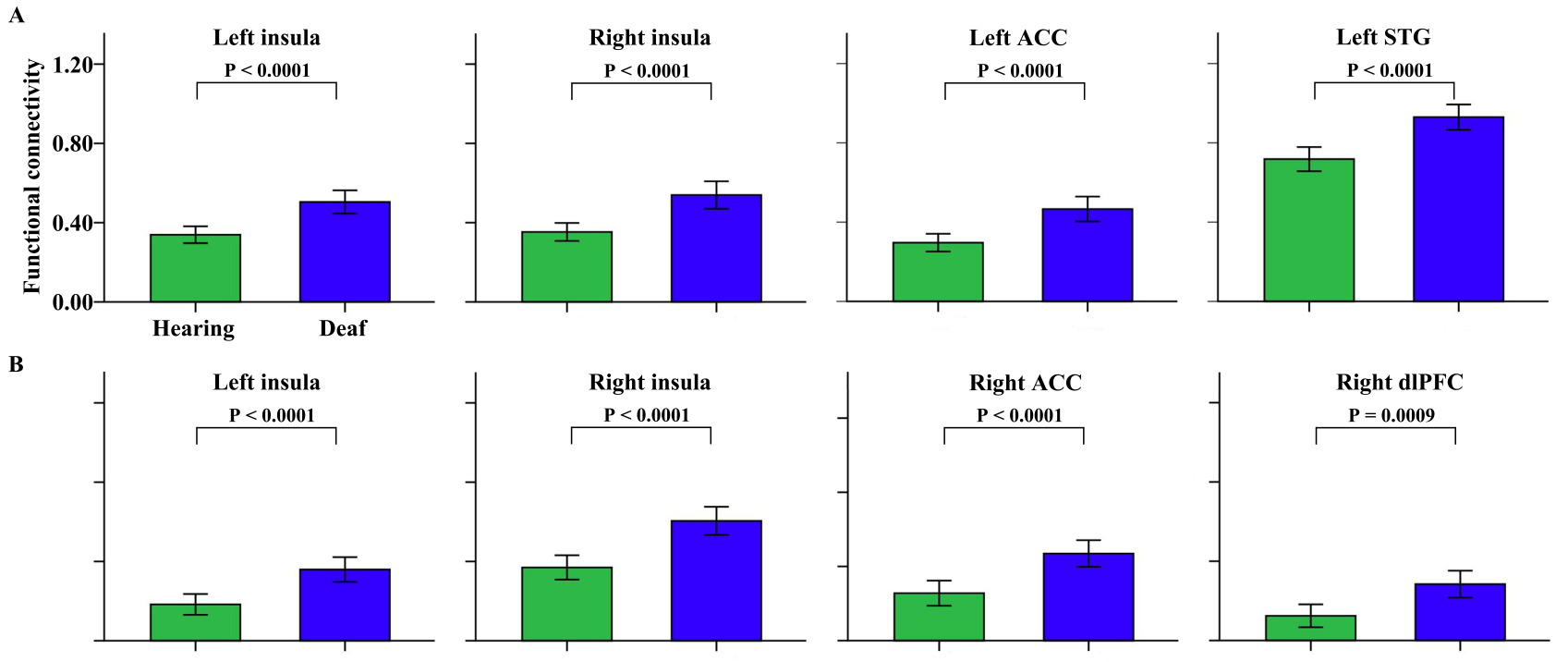


**Supplementary Figure S4**. Differences in functional connectivity of superior temporal gyrus without global signal regression between the early deaf and hearing subjects. Significant increased functional connectivity of left **(A)** and right superior temporal gyrus **(B)** were also observed in the early deaf compared with the hearing control.
